# Opposing effects of viral mediated brain expression of apolipoprotein E2 (apoE2) and apoE4 on apoE lipidation and Aβ metabolism in apoE4-targeted replacement mice

**DOI:** 10.1186/s13024-015-0001-3

**Published:** 2015-03-05

**Authors:** Jin Hu, Chia-Chen Liu, Xiao-Fen Chen, Yun-wu Zhang, Huaxi Xu, Guojun Bu

**Affiliations:** Fujian Provincial Key Laboratory of Neurodegenerative Disease and Aging Research, Institute of Neuroscience, School of Pharmaceutical Sciences, College of Medicine, Xiamen University, South Xiangan Road, Xiamen, Fujian 361102 China; Department of Neuroscience, Mayo Clinic, Jacksonville, FL 32224 USA

**Keywords:** Alzheimer’s disease, apoE, Lipidation, Aβ, apoE-TR mice

## Abstract

**Background:**

Human apolipoprotein E (apoE) exists in three major isoforms: apoE2, apoE3 and apoE4. In the brain, apoE is produced mostly by astrocytes and transports cholesterol to neurons via apoE receptors. Among the gene alleles encoding the three isoforms, the *APOE4* allele is the strongest genetic risk factor for late-onset Alzheimer’s disease (AD), whereas *APOE2* is protective. ApoE4 confers a gain of toxic function, a loss of neuroprotective function or a combination of both in AD pathogenesis. Given that therapeutic impacts of modulating apoE expression may be isoform-dependent, we sought to investigate the relationship between overexpressing apoE isoform and apoE-related functions in apoE-targeted replacement (TR) mice. Specifically, apoE isoform expression driven by the astrocyte-specific glial fibrillary acidic protein (GFAP) promoter was built into an adeno-associated virus serotype 8 (AAV8) vector and injected into the ventricles of postnatal day 2 (P2) apoE3-TR or apoE4-TR mice. Upon confirmation of apoE isoform expression, effects on apoE lipidation and the levels of amyloid-β (Aβ) in the brain were assessed.

**Results:**

AAV8-GFAP-apoE isoforms were specifically expressed in astrocytes throughout all brain regions, which led to overall increased apoE levels in the brain. Viral mediated overexpression of apoE4 in the apoE4-TR background increased poorly-lipidated apoE lipoprotein particles and decreased apoE-associated cholesterol in apoE4-TR mice. Conversely, apoE2 overexpression in apoE4-TR mice enhanced apoE lipidation and associated cholesterol. Furthermore, overexpression of apoE4 elevated the levels of endogenous Aβ, whereas apoE2 overexpression trended to lower endogenous Aβ.

**Conclusions:**

Overexpression of apoE isoforms induces differential effects in the apoE4-TR background: apoE4 decreases apoE lipidation and enhances Aβ accumulation, whereas apoE2 has the opposite effects. Our findings suggest that increasing apoE2 in *APOE4* carriers is a beneficial strategy to treat AD, whereas increasing apoE4 in *APOE4* carriers is likely harmful. We have also established novel methods to express apoE isoforms in mouse brain to study apoE-related pathways in AD and related dementia.

**Electronic supplementary material:**

The online version of this article (doi:10.1186/s13024-015-0001-3) contains supplementary material, which is available to authorized users.

## Background

Alzheimer’s disease (AD) is the leading cause of dementia in the elderly, which accounts for 60 to 80 percent of dementia cases [1]. Brain extracellular amyloid plaques and intracellular neurofibrillary tangles are the two pathological hallmarks of AD. Apolipoprotein E (apoE) is a major apolipoprotein and a cholesterol carrier in the brain [2]. The human *APOE* gene exists as three major polymorphic alleles: ε2, ε3 and ε4. Ample evidence indicates that the ε4 allele of the *APOE* gene is the strongest genetic risk factor for late-onset AD (LOAD), whereas the ε2 allele is protective [2-6].

While the primary function of apoE is to deliver cholesterol and other essential lipids to neurons through binding to cell surface apoE receptors, apoE also regulates brain metabolism of amyloid-β (Aβ) [7], accumulation of which leads to deposition of amyloid and is considered the initiating event in the pathogenesis of AD [8-10]. Previous studies have shown that brain Aβ levels and amyloid plaque loads are apoE isoform-dependent (E4 > E3 > E2) both in humans and in AD transgenic mouse models [11-13]. Several reports showed that the *APOE* genotype strongly affects apoE levels (E4 < E3 < E2) in human cerebrospinal fluid (CSF), brain parenchyma, and in apoE targeted-replacement (apoE-TR) mice [14-16]. Analysis on the levels of apoE and Aβ in different brain regions of non-demented individuals found that apoE levels negatively correlate with Aβ levels both in *APOE4* carriers and non-carriers [17]. ApoE protein levels in human CSF have also been shown to positively associate with CSF Aβ42 levels [14]. In addition, liver X receptors (LXRs) or the retinoid X receptor (RXR) agonists facilitate Aβ clearance and reverse the memory deficits in amyloid model mice by increasing apoE levels and its lipidation [18-20]. These results suggest that apoE levels and lipidation status likely contribute to Aβ clearance; thus, new therapeutic approaches aimed at increasing apoE expression are actively being pursued. However, to better design mechanism-based therapy, it is critical to understand how increasing apoE expression, in particular apoE4 in *APOE4* carriers, impacts apoE lipidation and Aβ metabolism.

Given that apoE is produced predominantly by astrocytes in the brain [21], we investigated the impact of overexpressing different human apoE isoforms in astrocytes in apoE-TR mice, in which the coding region of the mouse endogenous *Apoe* gene was replaced with one of the three human *APOE* alleles without changing the regulatory elements required for modulation of gene expression [22-24]. Thus, apoE-TR mice express human apoE isoforms at the physiological levels and respond to regulatory pathways in a physiological setting [22-25], providing an excellent *in vivo* system to explore the normal function of each *APOE* allele and apoE-associated diseases. Here, we used a gene delivery approach by which adeno-associated viral serotype 8 (AAV8) vectors expressing various human *APOE* alleles under the control of astrocyte-specific glial fibrillary acidic protein (GFAP) promoter were bilaterally injected into the cerebral lateral ventricles of neonatal apoE3-TR or apoE4-TR mice. We demonstrated that apoE isoforms are specifically expressed in astrocytes in the brain three months after injection. Importantly, we found that increasing apoE4 levels in apoE4-TR mice led to decreased apoE lipidation, lower apoE-associated cholesterol, and increased endogenous Aβ. Conversely, increasing apoE2 expression in apoE4-TR mice had the opposite, beneficial effects. Our findings for the first time reveal the consequential effects of overexpressing apoE isoforms in specific isoform background and should help to provide guidance for the designs of apoE-based targeted therapy to treat AD.

## Results

### Neonatal intracerebroventricular injection of AAV8-GFAP-apoE viruses results in sustained apoE isoform expression in astrocytes in mouse brain

**Figure 1:**
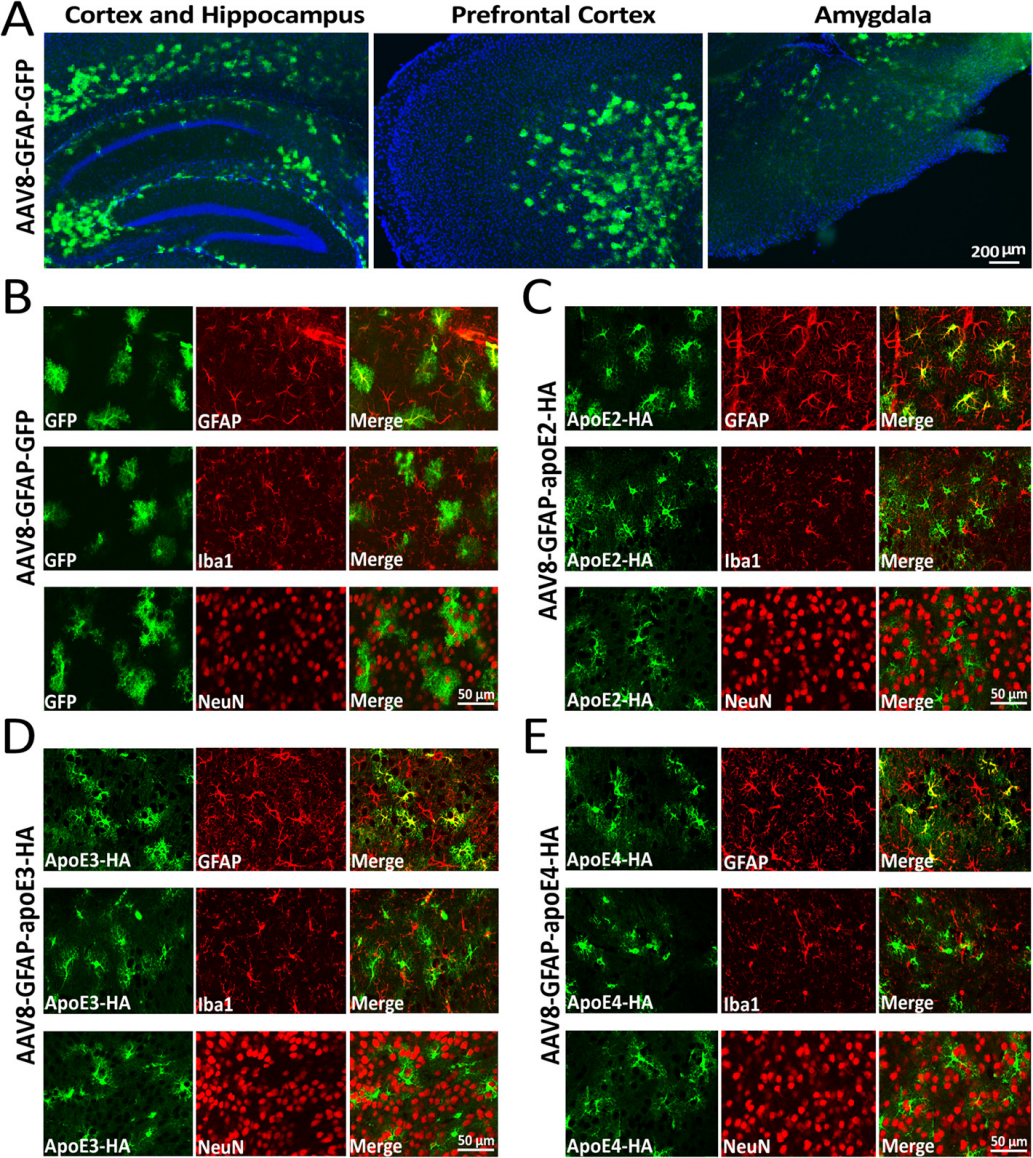
**Specific expression of AAV8-GFAP-GFP and AAV8-GFAP-apoE isoforms in astrocytes** ***in vivo.*** Newborn postnatal day 2 (P2) apoE3-TR mice were injected intracerebroventricularly with viruses carrying AAV8-GFAP-GFP or AAV8-GFAP-apoE isoforms. Expression of GFP or apoE isoforms was evaluated 3 months after viral injection. **(A)** GFP expression was detected by green fluorescence and the images from different brain regions are shown. **(B-E)** Mouse brain sections transduced with AAV8-GFAP-GFP (B), AAV8-GFAP-apoE2 **(C)**, AAV8-GFAP-apoE3 **(D)** or AAV8-GFAP-apoE4 **(E)** were co-immunostained with HA antibody for apoE expression and antibodies for cell type-specific markers (GFAP: astrocyte; Iba1: microglia; NeuN: neuron). Note that apoE (HA immunoreactivity) is specifically expressed in GFAP-positive astrocytes.

Intracerebroventricular injection of neonatal mice using AAV-based vectors is a relatively easy, fast and efficient way to modulate gene expression in the brain [26,27]. Previous studies have shown that when AAV8 viruses are injected intracranially after P0 of neonatal mice, viral mediated gene expression is detected primarily in astrocytes [27,28]. To specifically express apoE isoforms in astrocytes in mouse brain, we injected AAV8-GFAP viruses carrying cDNAs encoding GFP or one of three apoE isoforms into the cerebral ventricles of P2 neonatal apoE3-TR and apoE4-TR mice. An HA tag was included in apoE constructs to facilitate protein detection. Three months after virus injection, expression of GFP was evaluated in mouse brain. We found that intracranial delivery of AAV8-GFAP-GFP to neonatal mice resulted in an extensive transduction and GFP expression throughout the brain, including the cortex and hippocampus (Figure 1A). To evaluate cell-type specificity of AAV8-GFAP-mediated expression, we evaluated co-expression of GFP or apoE isoforms with cell-type specific markers for astrocytes, microglia, and neurons by immunofluorescence staining. We found that both GFP and apoE isoforms, detected with an HA antibody, were co-localized with GFAP-positive astrocytes, but not with Iba1-positive microglia or NeuN-positive neurons (Figure 1B-E). These results confirm that neonatal injection of AAV8-GFAP-apoE leads to sustained expression of apoE isoforms in astrocytes.

### AAV-mediated expression of apoE2 is significantly higher than those of apoE3 and apoE4

**Figure 2:**
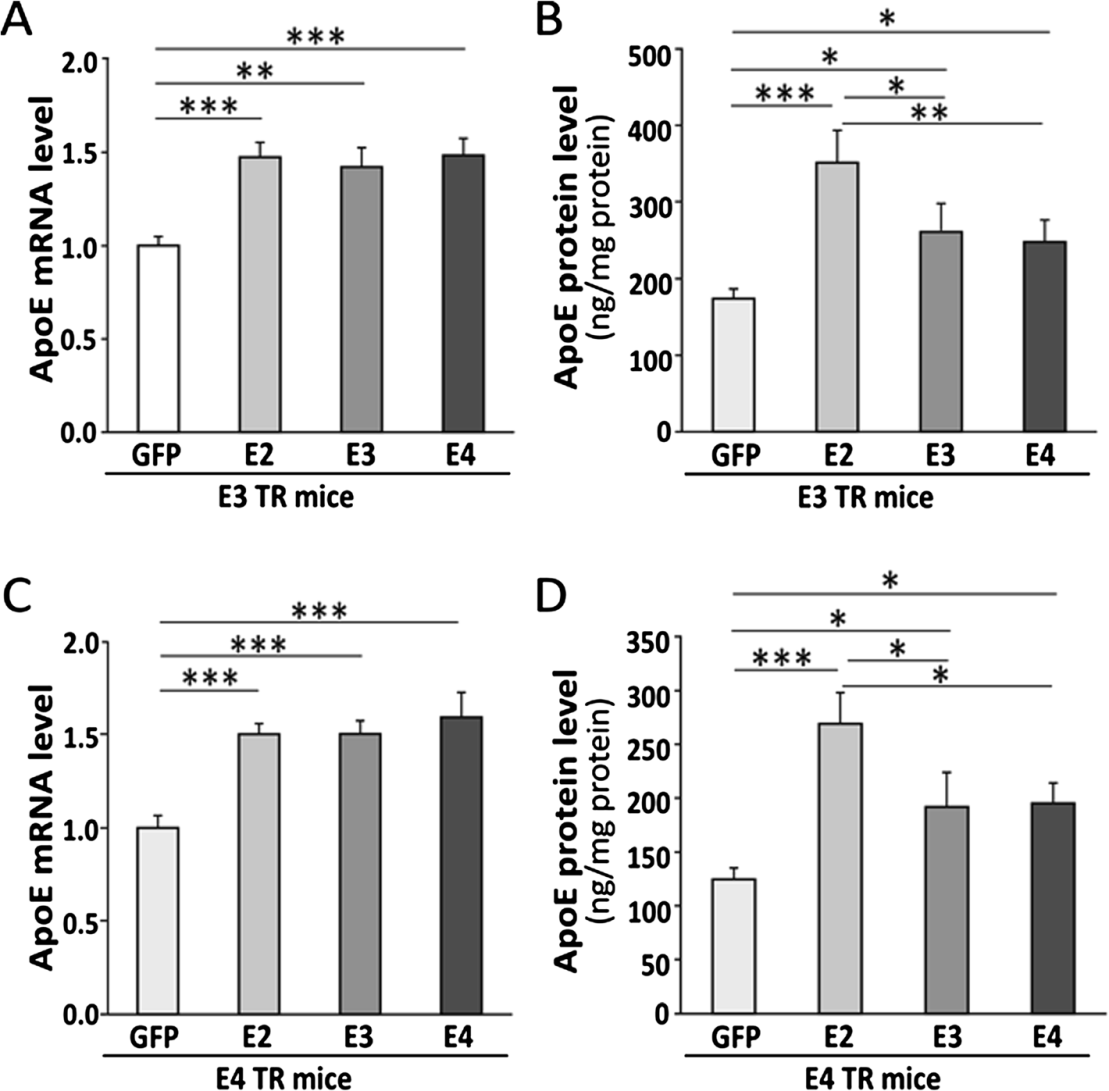
**Increased apoE expression in apoE-TR mice following AAV viral mediated expression of apoE isoforms in neonatal mice.** Neonatal P2 apoE3-TR or apoE4-TR mice were injected intracerebroventricularly with viruses carrying AAV8-GFAP-GFP or AAV8-GFAP-apoE isoforms, and mouse brains were harvested 3 months after viral injection. **(A** and **B)** Levels of apoE mRNA **(A)** and protein **(B)** in the cortices of apoE3-TR mice transduced with viruses encoding GFP or apoE isoforms were analyzed by real-time PCR or apoE-specific ELISA, respectively. (**C** and **D**) Same as in A and B, except apoE4-TR mice were used. Data are expressed as mean ± SEM (n = 7-8/group). *,p < 0.05; **,p < 0.01; ***,p < 0.001.

We next assessed the mRNA and protein levels of apoE in the cerebral cortex of apoE3-TR and apoE4-TR mice three months after injection with AAV8-GFAP-apoE isoforms or GFP control. Intracerebroventricular injection of AAV8-GFAP-apoE isoforms significantly increased apoE levels in the brains of apoE-TR mice compared with mice injected with GFP control viruses, as detected by real-time PCR (Figure 2A and C) and apoE ELISA (Figure 2B and D). While the apoE mRNA levels were not significantly different among apoE isoforms (Figure 2A and C), we found that the apoE protein levels are significantly higher in apoE-TR mice transduced with AAV8-GFAP-apoE2 viruses compared with those transduced with GFP, apoE3 or apoE4 viruses (Figure 2B and D). These results are consistent with a previous study [15] indicating that apoE2 is likely a more stable isoform compared with apoE3 and apoE4.

### Increasing apoE4 expression leads to decreased apoE lipidation in apoE4-TR mice

**Figure 3:**
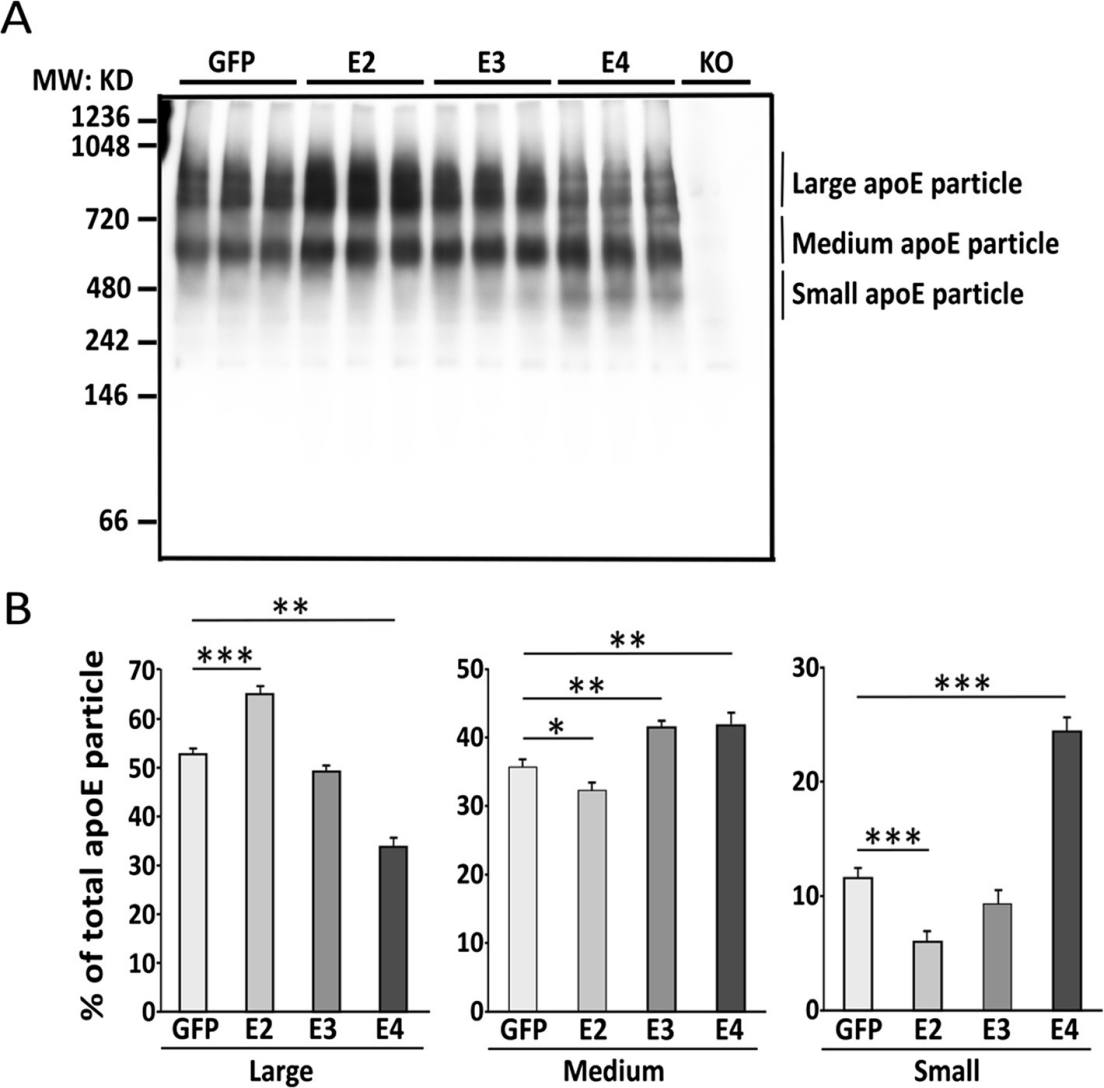
**Viral mediated expression of apoE4 in apoE4-TR mice shifts apoE particles to smaller sizes.** Neonatal P2 apoE4-TR mice were injected with AAV viruses and brains were harvested as in Figure 2. **(A)** ApoE-associated particles in the TBS fractions of cortical brain tissues were analyzed by native gel electrophoresis. ApoE particle-immunoreactivity was detected at approximately between 242 kDa and 1048 kDa, and defined as three categories of particle sizes: Large particles (>720 kDa); Medium particles (480–720 kDa) and Small particles (<480 kDa). Cortical lysates from age-matched *Apoe* knockout (KO) mice was used as a negative control for apoE immunoreactivity. **(B)** The percentages of apoE particles in different size categories were quantified. Data are expressed as mean ± SEM (n = 3-5). *,p < 0.05; **,p < 0.01; ***,p < 0.001.

ApoE lipidation is critical for its function in maintaining synapses [2,3,20]. Studies in humans [29] and in apoE-TR mice [30,31] suggest that apoE4 is significantly less lipidated than apoE2 and apoE3. To investigate the lipidation status of apoE in apoE-TR mice transduced with AAV8-GFAP-apoE isoforms, apoE-associated lipoprotein particles in the cortical brain lysates were evaluated by non-denaturing gel electrophoresis, followed by Western blotting (Figure 3). ApoE-immunoreactivity was detected in the molecular sizes between 242 and 1048 kDa. To quantify the distribution of apoE/lipoprotein particles, we defined particle sizes as large, medium and small (Figure 3A). Importantly, we found that the amounts of large apoE/lipoprotein particles were reduced and the small particles were increased in apoE4-TR mice transduced with AAV8-GFAP-apoE4 viruses compared with those transduced with GFP control (Figure 3B). In contrast, apoE4-TR mice transduced with AAV8-GFAP-apoE2 viruses exhibited increased amounts of large apoE particles and decreased amounts of small particles (Figure 3B). In like manner, transduction of AAV8-GFAP-apoE2 viruses in apoE3-TR mice increased the large particles, whereas transduction of AAV8-GFAP-apoE4 viruses increased the small particles (Additional file 1: Figure S1). These results indicate that apoE4 is associated with poorly and apoE2 abundantly lipidated particles.

**Figure 4:**
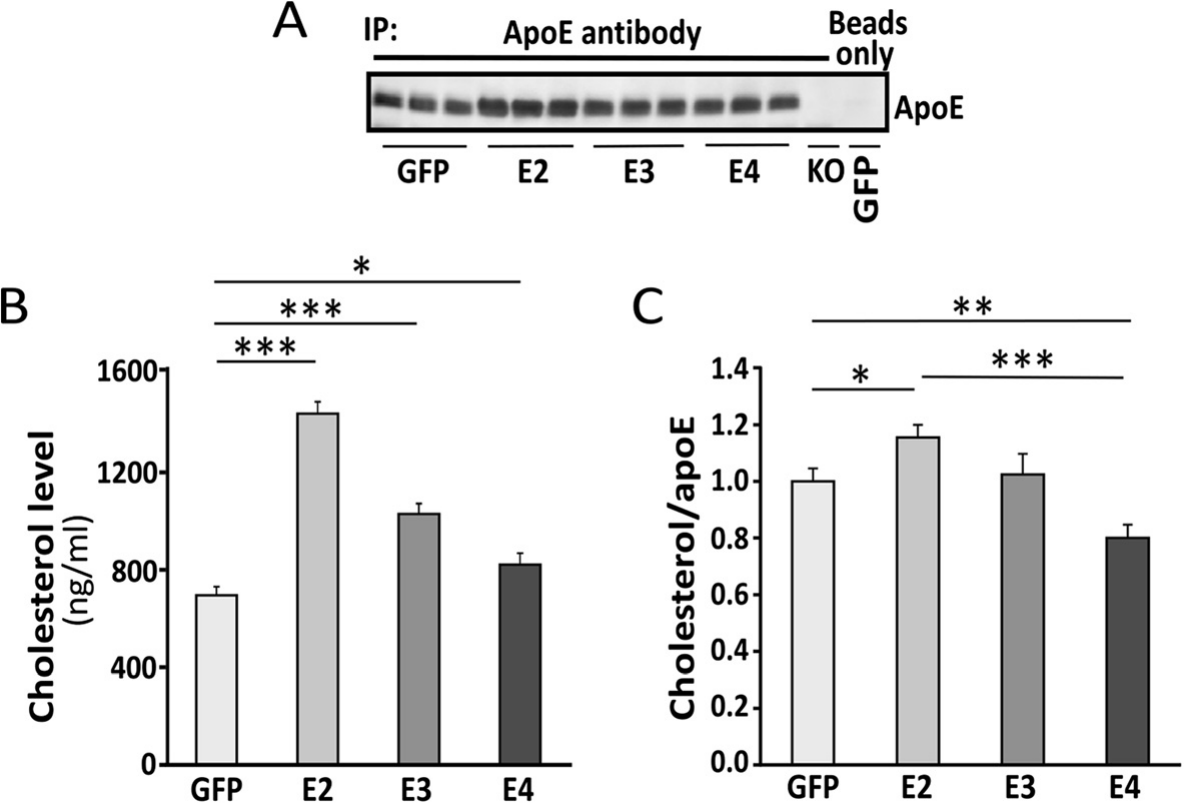
**Viral mediated expression of apoE4 in apoE4-TR mice leads to reduced levels of apoE-associated cholesterol.** Neonatal P2 apoE4-TR mice were injected with AAV viruses and brains were harvested as in Figure 2. Mouse cortical brain tissues were lysed in TBS and the apoE-associated particles were isolated by immunoprecipitation using a biotinylated-apoE specific antibody. Mice injected with GFP viruses treated with beads only were used as a negative control. **(A)** The immunoprecipitated apoE under different conditions was detected by Western blot. **(B)** ApoE-associated cholesterol was analyzed by Amplex Red Cholesterol Assay. **(C)** The ratios of cholesterol-to-apoE under each condition were calculated. Data are expressed as mean ± SEM (n = 3-5/group). *,p < 0.05; **,p < 0.01; ***,p < 0.001.

To further evaluate apoE lipidation status, we next quantified the amounts of cholesterol associated with apoE isoforms in the buffer/TBS-soluble fractions of apoE3-TR and apoE4-TR mice transduced with different viruses. The apoE-containing particles were immunoprecipitated with a biotinylated apoE antibody and the amounts of cholesterol co-immunoprecipitated with apoE were quantified. Transduction of AAV8-GFAP-apoE isoforms increased the levels of apoE and apoE-associated cholesterol in both apoE3-TR mice (Additional file 1: Figure S2A and B) and apoE4-TR mice compared with those transduced with GFP control (Figure 4A and B). Interestingly, mice transduced with apoE2 had the highest apoE and cholesterol levels (Figure 4A and B; Additional file 1: S2A and B). In addition, the cholesterol-to-apoE ratio was significantly increased in apoE4-TR mice transduced with apoE2, while it was decreased among apoE4-TR mice transduced with apoE4 (Figure 4C). We did not observe a significant difference in the ratio of cholesterol-to-apoE in apoE3-TR mice transduced with AAV8-GFAP-apoE isoforms compared with controls (Additional file 1: Figure S2C). Further, there were no significant differences in the levels of ATP-binding cassette transporters A1 (ABCA1) and ATP-binding cassette transporters G1 (ABCG1), two major cholesterol transporters, in apoE3-TR or apoE4-TR mice transduced with AAV8-GFAP-apoE isoforms compared with controls (Additional file 1: Figure S3). These results indicate that AAV8-mediated transduction of apoE in astrocytes increases apoE-associated cholesterol in the brains in an isoform-dependent manner.

### ApoE4 overexpression in the brain of apoE4-TR mice results in increased Aβ accumulation

**Figure 5:**
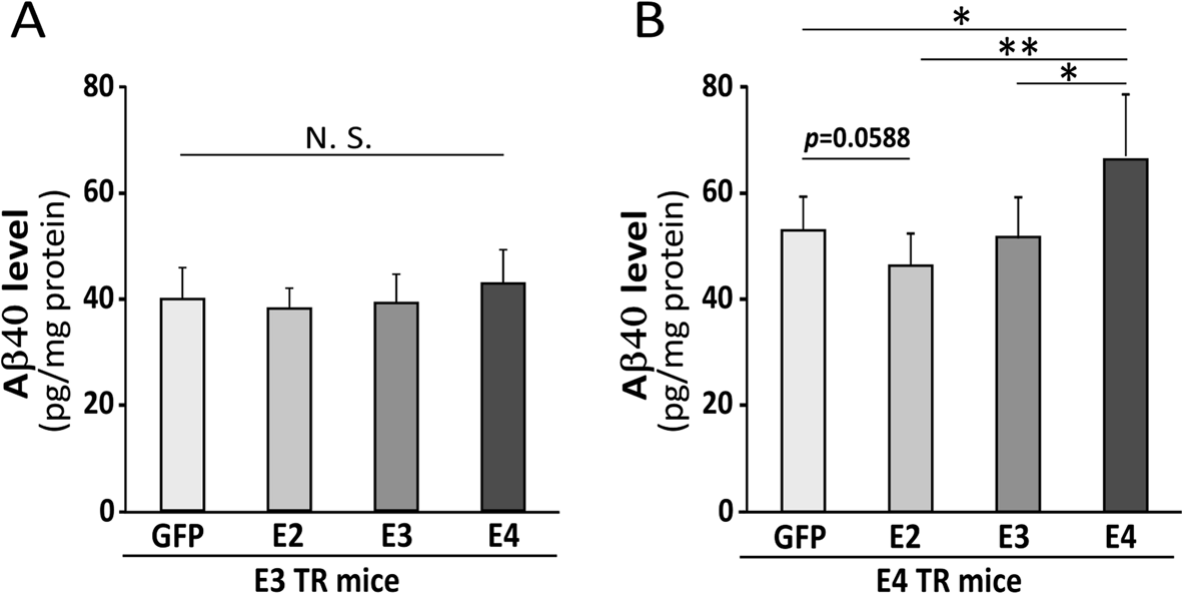
**Viral mediated expression of apoE4 in apoE4-TR mice leads to increases of mouse endogenous Aβ.** Neonatal P2 apoE3-TR or apoE4-TR mice were injected with AAV viruses and brains were harvested as in Figure 2. **(A** and **B)** Levels of mouse endogenous Aβ in the cortices of apoE3-TR mice **(A)** or apoE4-TR mice **(B)** transduced with AAV viruses encoding GFP or apoE isoforms were quantified by mouse Aβ-specific ELISA. Data are expressed as mean ± SEM (n = 7-8/group). N.S., Not significant; *,p < 0.05; **,p < 0.01.

**Figure 6:**
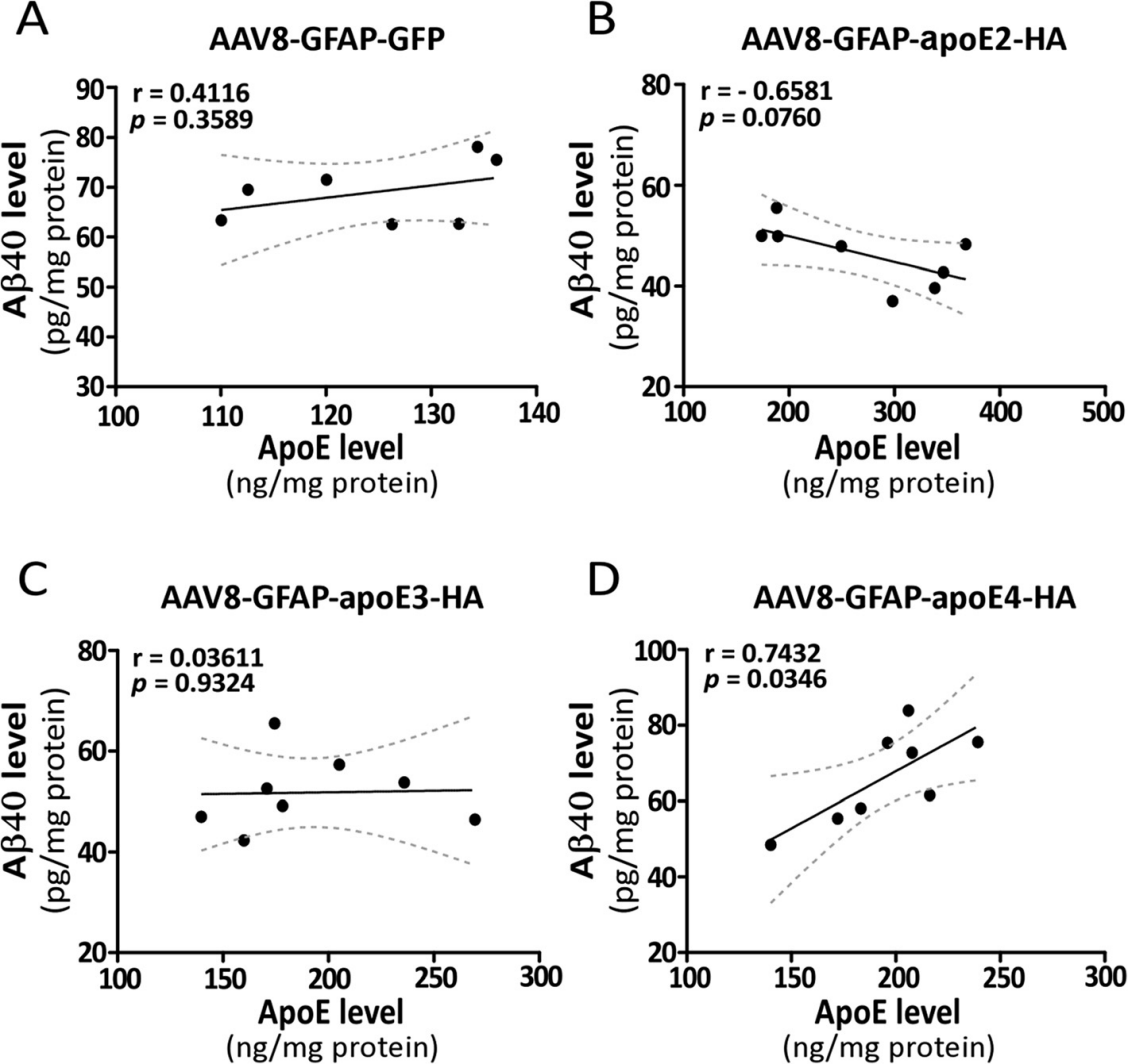
**Positive correlations between mouse endogenous Aβ and apoE in apoE4-TR mice transduced with AAV8-GFAP-apoE4 viruses.** Three months after intracranial delivery of viruses carrying AAV8-GFAP-GFP or AAV8-GFAP-apoE isoforms, the levels of mouse endogenous Aβ40 and apoE in the cortices of apoE4-TR mice were examined by Aβ40-specific ELISA and apoE-specific ELISA, respectively. **(A-D)** The correlation between levels of Aβ40 and apoE in the cortex of apoE4-TR mice transduced with GFP **(A)**, apoE2 **(B)**, apoE3 **(C)** or apoE4 **(D)** were analyzed and plotted. The Pearson correlation coefficient (r) and p values are shown.

Mounting evidence indicates that apoE regulates Aβ metabolism, aggregation and deposition in an isoform-dependent manner [7,32] and that Aβ clearance is slower in apoE4-TR mice compared with apoE3-TR mice [12]. In addition, many studies suggest that lipid-poor apoE promotes Aβ aggregation while fully lipidated apoE favors Aβ clearance [20,26,27]. In this study, we found that AAV8-mediated transduction of apoE in astrocytes regulated apoE lipidation in an isoform-dependent manner. We thus examined the differential effects of increasing apoE isoforms on Aβ metabolism in the brains of apoE3-TR and apoE4-TR mice. Transduction of various apoE isoforms in apoE3-TR mice did not significantly impact the levels of endogenous Aβ40 in the cortex (Figure 5A). However, when apoE isoforms were overexpressed in the background of apoE4-TR mice, apoE4 significantly increased the levels of endogenous Aβ40 whereas apoE2 trending to the opposite (Figure 5B). We further performed correlation analyses between the levels of apoE and endogenous Aβ40 in apoE4-TR mice transduced with different viruses (Figure 6A-D) and found that the levels of endogenous Aβ40 positively correlated with those of apoE in apoE4-TR mice transduced with AAV8-GFAP-apoE4 viruses (Figure 6D). In contrast, there was a trend of negative correlation between the levels of Aβ40 and apoE in apoE4-TR mice transduced with AAV8-GFAP-apoE2 viruses (Figure 6B). There were no significant correlations between the levels of endogenous Aβ40 and apoE in apoE3-TR mice transduced with viruses expressing different apoE isoforms (Additional file 1: Figures S4A-D). To determine whether transduction of AAV8-GFAP-apoE viruses affects the processing of amyloid precursor protein (APP) in mouse brains, we analyzed the levels of full-length APP, APP α- and β-C-terminal fragments (α-CTFs and β-CTFs) in apoE3-TR and apoE4-TR mice. Transduction of AAV8-GFAP-apoE viruses did not significantly affect the levels of full-length APP, APP α-CTF or β-CTFs in apoE3-TR or apoE4-TR mice compared with those transduced with GFP control viruses (Additional file 1: Figure S5A and B), suggesting that overexpression of apoE isoforms does not affect APP expression or processing. Together, these results suggest that increasing the expression level of apoE4 is likely detrimental, while apoE2 is beneficial, in regard to brain Aβ accumulation.

## Discussion

The most prevalent genetic risk factor for late-onset AD is the *APOE* genotype with an allele-specific risk profile: *APOE4* > *APOE3* > *APOE2* [33,34]). Despite progress in the past two decades [2,3], it remains unclear how apoE4 increases, and apoE2 decreases, the risk of AD. The amyloid hypothesis proposes that an imbalance between Aβ production and clearance leads to Aβ accumulation in the brain that initiates AD pathogenesis [8,10]. Multiple lines of evidence demonstrate that apoE isoforms differentially regulate Aβ metabolism in the brain [35-38], likely through modulation of Aβ clearance and aggregation [3,12,32]. These findings suggest that apoE levels, lipidation status and isoform-dependent effects may differently impact Aβ metabolism, thereby contributing to or preventing AD pathogenesis.

In the current study, we used AAV8 constructs driven by an astrocyte-specific GFAP promoter to express three apoE isoforms (AAV8-GFAP-apoE2/3/4) in neonatal apoE3-TR or apoE4-TR mice through intracranial injection. Transduction of AAV8-GFAP-apoE led to sustained expression of apoE isoforms in mouse brain. We found that transduction of apoE4 in apoE4-TR background significantly increased the amounts of small, lipid-poor apoE particles and the levels of endogenous Aβ. Conversely, transduction of apoE2 in the apoE4-TR background led to an increase in the amounts of large, lipid-rich apoE particles and decreased endogenous Aβ. Our findings indicate that modulating the levels of apoE isoforms had differential effects on the lipidation status of apoE and Aβ levels in apoE4-TR, less so in apoE3-TR mice. Specifically, overexpression of apoE2 and apoE4 in apoE4 background leads to opposite effects on the amounts of cholesterol associated with apoE and Aβ accumulation with apoE4 being detrimental and apoE2 beneficial. We did not observe significant changes in APP proteolytic processing products in mice transduced with different apoE isoforms, indicating that APP processing to Aβ is not regulated by the expression levels of apoE isoforms.

A recent study assessed the effects of overexpressing apoE isoforms in the background of amyloid model mice with endogenous murine apoE background [39]. They found that overexpression of apoE4 leads to higher levels of Aβ and enhanced amyloid deposition, whereas overexpression of apoE2 had the opposite effects. Our current study uniquely address how overexpression of apoE isoforms in different human apoE isoform background impact apoE lipidation and Aβ accumulation, events that are critical for the synaptic functions and the pathogenesis of AD. When combined, these studies should provide mechanism-based guidance on regulating apoE expression and lipidation in the designs of therapeutic approaches to treat AD.

The mechanism underlying the protective effect of apoE2 against AD remains unclear. Our results show that apoE protein levels are significantly higher in mice transduced with apoE2 compared with mice transduced with GFP controls, apoE3 or apoE4 viruses, despite similar mRNA levels. Previous studies have shown that apoE2 may exhibit a more stable conformation [40] and has lower affinity to the low-density lipoprotein (LDL) receptor, which may lead to decreased catabolism [15]. Thus, the higher levels of apoE observed in apoE2-transduced mice may be attributed to its increased stability and decreased turnover. Previous studies suggest that formation of protein complexes between apoE and Aβ may facilitate Aβ clearance [41]. Compared with apoE2 or apoE3, apoE4/Aβ complexes have been shown to be less stable, which is likely attributed to the poor lipidation status of apoE4/lipoprotein particles [7,31,42]. In addition, complex formation between Aβ and apoE isoforms may affect their binding property to their individual or common receptors including the low-density lipoprotein receptor-related protein 1 (LRP1), thereby further affects Aβ clearance [32,43]. Thus, it is possible that overexpression of apoE2 increases and apoE4 decreases apoE/Aβ complex formation and therefore, the clearance of Aβ in the brain.

Emerging evidence indicates that apoE lipidation status directly affects its role in Aβ clearance [20,44,45]. Our current study demonstrates that AAV-mediated transductions of apoE isoforms have different effects on the lipidation status of apoE. Compared with controls, transduction of apoE4 in apoE4-TR mice increased the proportion of poorly-lipidated apoE. Several studies suggest that apoE4 is significantly less lipidated than apoE2 and apoE3 in both human and APP transgenic mice expressing human apoE isoforms [29-31]. ApoE4 more easily self-aggregates, which may affect its lipidation capacity [46]. Hence, it is possible that overexpression of apoE4 in apoE4-TR mice results in apoE4 aggregation, which might impair its lipidation. Consistent with this notion, haploinsufficiency of ABCA1, which mediates apoE lipidation, impairs Aβ clearance and exacerbates amyloid deposition in apoE4-TR mice, but not in apoE3-TR mice [47]. Though we did not observe an alteration in the levels of ABCA1 and ABCG1, boosting apoE4 in apoE4-TR mice may enhance apoE4 aggregation in parallel with an impairment of its lipidation, which in turn decreases Aβ clearance and/or promotes Aβ deposition.

In addition to its effects on Aβ metabolism, apoE mediates neuronal delivery of cholesterol, an essential component for synaptic plasticity required for learning and memory formation [2]. Given the ongoing clinical trial aimed at increasing apoE levels and its lipidation [20], it is imperative to understand the consequences of boosting apoE on cholesterol homeostasis and Aβ metabolism in the background of different apoE isoforms. Our current study directly demonstrates the divergent effects of apoE2 and apoE4 isoforms on Aβ accumulation and apoE lipidation status, effects that are more pronounced in apoE4-TR mice.

## Conclusions

Here, we report that increasing apoE4 in the brain of apoE4-TR mice reduces lipid-rich apoE and promotes Aβ accumulation, while increasing apoE2 in the brain leads to opposite effects. To the best of our knowledge, this is the first report demonstrating the impact of boosting different apoE isoforms in astrocytes on cholesterol and Aβ metabolism in the brain of apoE3-TR or apoE4-TR mice. Our findings suggest that astrocyte expression of apoE2 isoforms through AAV-mediated transduction or delivery of apoE2 mimetic into the brain might have therapeutic value, while increasing apoE4 in the brain may be harmful, especially for carriers of the *APOE4* allele.

## Materials and methods

### Animals

Human apoE3-TR or apoE4-TR mice, which express respective human apoE isoform driven by the endogenous murine *ApoE* promoter, were obtained from Taconic. All animal experiments were conducted in compliance with the protocols of the Institutional Animal Care and Use Committee at Mayo Clinic.

### Viral vector construction and production

The AAV8 vector construction and production were performed by the Hope Center Viral Vectors Core at the Washington University School of Medicine. ApoE2, 3, or 4 cDNA in pcDNA3.1 was amplified by PCR with the forward primer containing a BamHI site and the reverse primer containing a SalI site and an influenza hemagglutinin (HA) tag sequence. The PCR products were digested with BamHI and SalI and inserted into an AAV8-GFAP-GFP backbone in which the GFP was replaced with the PCR products. The resulting AAV8-GFAP-aopE2-HA, AAV8-GFAP-aopE3-HA, and AAV8-GFAP-aopE4-HA contain the respective apoE isoforms with a HA tag under the control of a GFAP promoter. The AAV8 viruses were produced as previously described [48]. Briefly, the packaging cell line HEK293 is maintained in Dulbecco’s modified Eagles medium (DMEM), supplemented with 5% fetal bovine serum (FBS), 100 units/ml penicillin, 100 μg/ml streptomycin in 37°C incubator with 5% CO_2_. The cells were co-transfected with pAAV2/8, pHelper, and rAAV transfer plasmid containing GFP or apoE isoforms using the calcium phosphate precipitation method. The cells were incubated at 37°C for 3 days before harvesting. Cells were lysed by three freeze/thaw cycles. The cell lysate were treated with 50U/ml of Benzonaze followed by iodixanol gradient centrifugation. The iodixanol gradient fraction is further purified by HiTrap Q column chromatography (GE Healthcare) and concentrated with Vivaspin concentrator (Bohemia, NY). The virus titer was determined by dot blot assay.

### Intracranial injections in neonatal mice

AAV8 virus intracranial injection in neonatal mice was performed as previously described with minor modifications [27,28]. Briefly, postnatal day 2 (P2) mice were cryoanesthetized on ice for 5 min and injected intracerebroventricularly with 2 μl (~10^13^ viral particles/ml) of viruses into both hemispheres using a syringe (7642–01, Hamilton) with a 30 gauge needle (7803–07, Hamilton). After injections were complete, pups were placed on a warming pad until they regained normal color and resumed movement. All injected animals were then returned to their mothers for further recovery.

### Preparation of mouse brains

Three months post AAV virus injection, mice were anesthetized using isoflurane, perfused with PBS, and their brains were rapidly harvested. The right hemibrains were fixed in 4% paraformaldehyde and used for immunofluorescence analysis, and left hemibrains were dissected and kept frozen at −80°C until further biochemical analysis.

### Immunohistochemistry

The immunohistochemistry for the brain sections was performed as previously described [27]. Briefly, brains were fixed overnight at 4°C in fresh 4% paraformaldehyde and then transferred to 30% sucrose for cryoprotection. Brains were frozen on dry ice and then sectioned at 45 μm using a freezing-sliding microtome (LEICA SM2400, Leica). Sections were permeated and blocked with 3% goat serum containing 0.1% Triton-X 100 in Tris-buffered saline (TBS) buffer (25 mM Tris, 0.15 M NaCl, pH 7.4) for 1 h at room temperature. Then, the sections were incubated with mouse anti-HA (1:1000, Covance), rabbit anti-GFAP (1:1000, Millipore), rabbit anti-NeuN (1:1000, Millipore) or rabbit anti-Iba1 (1:1000, Wako Chemicals) antibodies in blocking solution at 4°C overnight. The sections were washed with TBS/0.5% Tween 20 (TBST) and incubated with Alexa Fluor 488-conjugated goat anti-mouse (1:400; Invitrogen) and Alexa Fluor 568-conjugated donkey anti-rabbit (1:400; Invitrogen) secondary antibodies for 2 h at room temperature. Images were obtained by confocal microscope (LSM 510 META, Zeiss) and analyzed by ZEN software.

### Quantitative real-time PCR

Total RNAs were extracted from frozen cortical tissues using Trizol (Invitrogen) and Direct-zol RNA MiniPrep kit (Zymo Research). Reverse transcription was performed using SuperScript III First-Strand Synthesis System (Invitrogen). Real-time qPCR was conducted with Universal SYBR Green Supermix (Bio-Rad) using an iCycler thermocycler (Bio-Rad). The following primers were used: Human apoE mRNA forward primer, TGTCTGAGCAGGTGCAGGAG; and reverse primer, TCCAGTTCCGATTTGTAGG. Mouse β-actin forward primer, AGTGTGACGTTGACATCCGTA; and reverse primer, GCCAGAGCAGTAATCTCCTTC Relative mRNA levels were calculated by ΔΔCt method with β-actin used as a reference.

### ELISA for human apoE

Cortical tissues were homogenized with a Polytron homogenizer in ice cold TBS buffer containing protease inhibitor and phosphate inhibitor cocktails (Roche). The homogenates were centrifuged at 100,000 g for 30 min at 4°C and the supernatants were collected. ApoE levels were analyzed using an ELISA. Briefly, 96-well plates were coated overnight with an apoE antibody (AB947, Millipore) in carbonate buffer at 4°C overnight. The plates were blocked with 1% Block Ace in PBS, and then washed with PBS 3 times. Recombinant apoE (Fitzgerald) along with samples were diluted and added at a volume of 100 μl/well incubated at 4°C overnight. The plates were washed and incubated with biotin-conjugated goat anti-apoE antibody (Meridian Life Science) for 2 h at room temperature. After incubation with Horseradish Peroxidase Avidin D (Vector Laboratories) for 90 min at room temperature, the plate was developed by adding tetramethylbenzidine Super Slow substrate (Sigma). The reaction was stopped and read at 450 nm with a microplate reader.

### ELISA for mouse endogenous Aβ

Mouse endogenous Aβ was extracted from cortical tissues by diethylamine as previously described [49]. The Aβ levels in age-matched APP knockout mice were used as a negative control and subtracted as background. For detection of endogenous mouse Aβ40, 96-well plates were coated at 4°C overnight with 13.1.1 mAb (anti-Aβ35-40) [49,50]. The plates were blocked with 1% Block Ace in PBS, washed with PBS and loaded with samples. Synthetic rodent Aβ40 (AnaSpec) was used as standards. Following overnight incubation at 4°C, the plates were washed, followed by incubation with HRP-conjugated mAb 32.4.1 (rodent Aβ1-16 specific) [49] for detection. The plates were developed with tetramethylbenzidine Supersensitive substrate (Sigma). The reaction was stopped and read at 450 nm with a microplate reader.

### Analyses of apoE/lipoprotein particles by Blue Native PAGE and Western blot

Cortical tissues were homogenized with a Polytron homogenizer in ice cold TBS buffer containing protease inhibitor and phosphate inhibitor cocktails (Roche). Proteins were separated by Native PAGE™ Novex 4–16% Bis-Tris gels (Invitrogen) under native conditions following the manufacturer’s instructions and transferred to PVDF (Millipore) at 100 V for 1 h using the Trans-Blot Cell (Bio-Rad). Blots were treated with Ponceau S Staining Solution (0.1% (w/v) Ponceau S in 5% (v/v) acetic acid) to visualize the molecular mass markers. The NativeMark Unstained Protein Standard from Invitrogen was used for estimation of particle sizes. After washing in TBS, blots were processed for Western blot. The membrane was incubated with goat anti-apoE antibody (K74180B, Meridian Life Science) overnight at 4°C, followed by peroxidase-labeled donkey anti-goat antibody (Santa Cruz). The membrane was developed with Lumigen ECL Ultra Western Blotting HRP Substrate (Lumigen), and the signals were detected by Fuji film Luminescent Image Analyzer (LAS4000). An antibody that recognizes the C-terminus of APP (18961, IBL-America) was used for detecting APP and its C-terminal fragments. Anti-β-actin (Sigma) was used as a loading control. Western blot bands were quantified by Image J software.

### Immunoprecipitation (IP) of apoE particles and cholesterol assay

Streptavidin-conjugated agarose beads (Sigma) were first incubated with biotin-conjugated goat anti-apoE antibody (K74180B, Meridian Life Science) for 2 h at room temperature with shaking. The unbound antibodies were removed by washing with TBS three times. The antibody-bound agarose beads were then mixed with brain lysates in TBS fractions (500 μg total protein) at 4°C overnight. The IP solution was centrifuged for 2 min at 1,000 g to collect agarose beads. The beads were washed with cold TBS three times and re-suspended with TBS/0.1% Triton X-100. The resulting suspension was subjected to Western blot analysis in which equal volumes of samples were loaded. Proteins were separated by 4-20% Mini-PROTEAN TGX Gel (Bio-Rad) and transferred to Immobilon-P PVDF (Millipore). The membranes were incubated with apoE antibody (K74180B, Meridian Life Science), followed by IRDye secondary antibody (LI-COR Biosciences). The results were visualized and quantified by Odyssey infrared imaging system (LI-COR Biosciences). The remaining suspension was used for the detection of apoE particle-associated cholesterol quantified by Amplex Red cholesterol assay (Invitrogen) according to the manufacturer’s protocol.

### Statistical analysis

All data were analyzed by one-way analysis of variance (ANOVA) with a Tukey’s post-hoc test using GraphPad Prism 5. Data were presented as average ± SEM. A p value of < 0.05 was considered statistically significant.

## Additional file

Additional file 1: Figure S1. Analysis of apoE-associated lipoprotein particles in apoE3-TR mice transduced with viruses carrying AAV8-GFAP-apoE isoforms. **Figure S2.** ApoE-associated cholesterol levels in apoE3-TR mice transduced with viruses carrying AAV8-GFAP-apoE isoforms. **Figure S3.** Overexpression of apoE isoforms does not affect the levels of ABCA1 and ABCG1 in apoE-TR mice. **Figure S4.** Correlation analysis between mouse endogenous Aβ and apoE in apoE3-TR mice transduced with viruses carrying AAV8-GFAP-apoE isoforms. **Figure S5.** APP expression and processing are not altered in apoE-TR mice upon overexpression of apoE isoforms.

## Abbreviations

AD: Alzheimer’s disease
ApoE: Apolipoprotein E
AAV8: Adeno-associated virus serotype 8
GFAP: Astrocyte-specific glial fibrillary acidic protein (GFAP)
Aβ: Amyloid-β
apoE-TR mice: apoE targeted-replacement mice
RXR: Retinoid X receptor
CSF: Cerebrospinal fluid
APP: Amyloid precursor protein
ABCA1: ATP-binding cassette transporters A1
