# Supplementary material for: Opposing effects of viral mediated brain expression of apolipoprotein E2 (apoE2) and apoE4 on apoE lipidation and Aβ metabolism in apoE4-targeted replacement mice
Source: Mol Neurodegener. 2015 Mar 5;10:6. doi: 10.1186/s13024-015-0001-3 (PMC4356137; doi:10.1186/s13024-015-0001-3)
Supplement: Additional file 1: Figure S1. — Analysis of apoE-associated lipoprotein particles in apoE3-TR mice transduced with viruses carrying AAV8-GFAP-apoE isoforms. Figure S2. ApoE-associated cholesterol levels in apoE3-TR mice transduced with viruses carrying AAV8-GFAP-apoE isoforms. Figure S3. Overexpression of apoE isoforms does not affect the levels of ABCA1 and ABCG1 in apoE-TR mice. Figure S4. Correlation analysis between mouse endogenous Aβ and apoE in apoE3-TR mice transduced with viruses carrying AAV8-GFAP-apoE isoforms. Figure S5. APP expression and processing are not altered in apoE-TR mice upon overexpression of apoE isoforms. [file 13024_2015_1_MOESM1_ESM.pdf]

Additional figures

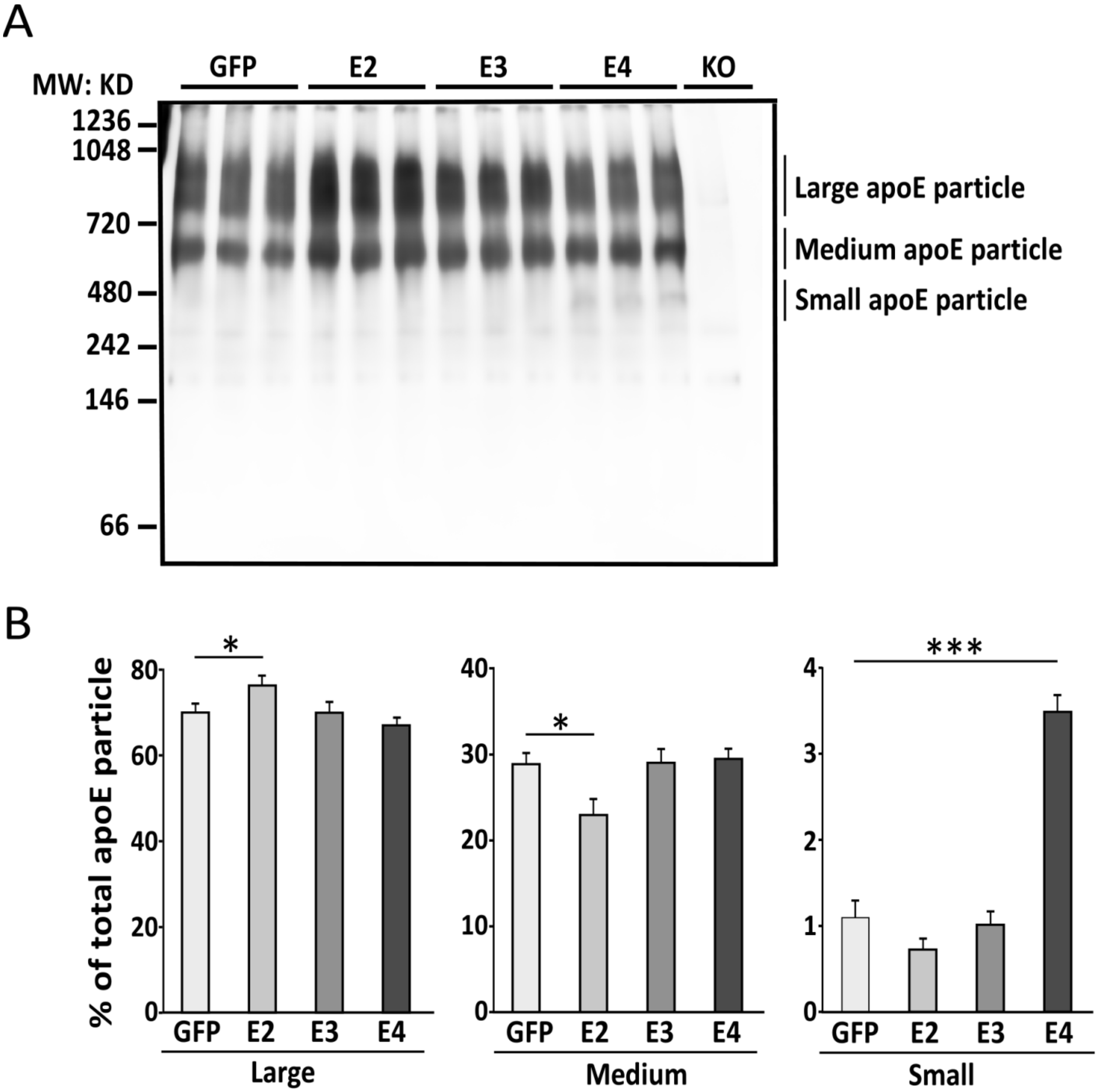

Figure S1. Analysis of apoE-associated lipoprotein particles in apoE3-TR mice transduced with viruses carrying AAV8-GFAP-apoE isoforms

Neonatal P2 apoE3-TR mice were injected with AAV viruses and brains were harvested as in Figure 2. (A) ApoE-associated particles in the TBS fractions of cortical brain tissues were analyzed by native gel electrophoresis. ApoE lipoprotein particles were defined as three categories of particle sizes: Large particles (>720 kDa); Medium particles (480-720 kDa) and Small particles (<480 kDa). Cortical lysates from age-matched *Apoe* knockout mice was used as a negative control for apoE immunoreactivity. (B) The percentages of apoE particles in different size categories were quantified. Data are expressed as mean ± SEM (n=3-5). *, $p < 0.05$; **, $p < 0.01$; ***, $p < 0.001$.

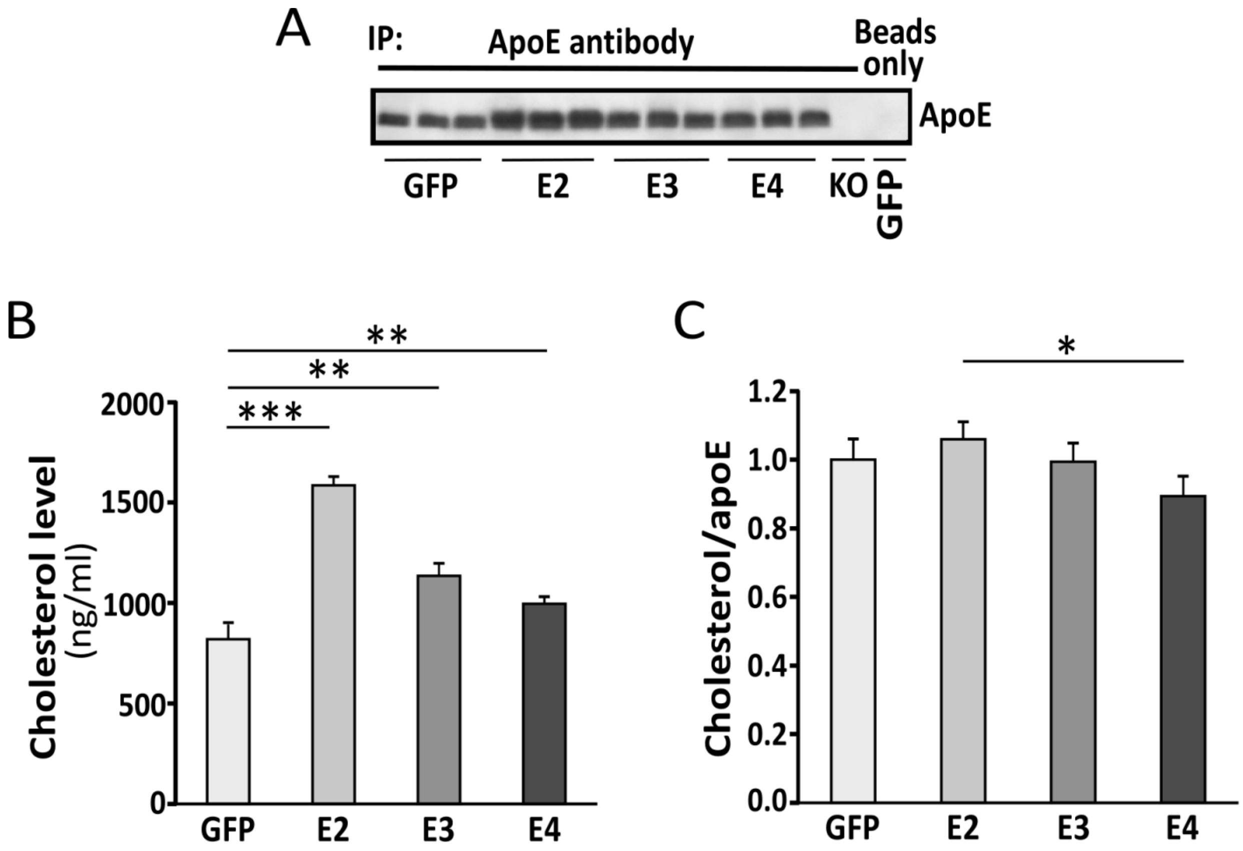
A
IP:
ApoE antibody
Beads only
ApoE
GFP
E2
E3
E4
KO
GFP
B
**
**
***
2000
1500
1000
500
0
Cholesterol level (ng/ml)
GFP
E2
E3
E4
C
*
1.2
1.0
0.8
0.6
0.4
0.2
0.0
Cholesterol/apoE
GFP
E2
E3
E4

Figure S2. ApoE-associated cholesterol levels in apoE3-TR mice transduced with viruses carrying AAV8-GFAP-apoE isoforms

Neonatal P2 apoE3-TR mice were injected with AAV viruses and brains were harvested as in Figure 2. Mouse cortical brain tissues were lysed in TBS and the apoE-associated particles were isolated by immunoprecipitation using a biotinylated-apoE specific antibody. Mice injected with GFP viruses treated with beads only were used as a negative control. (A) The immunoprecipitated apoE under different conditions was detected by Western blot. (B) ApoE-associated cholesterol was analyzed by Amplex Red Cholesterol Assay. (C) The ratios of cholesterol to apoE under each condition were calculated. Data are expressed as mean ± SEM (n=3-5/group). *, $p < 0.05$; **, $p < 0.01$; ***, $p < 0.001$.

A

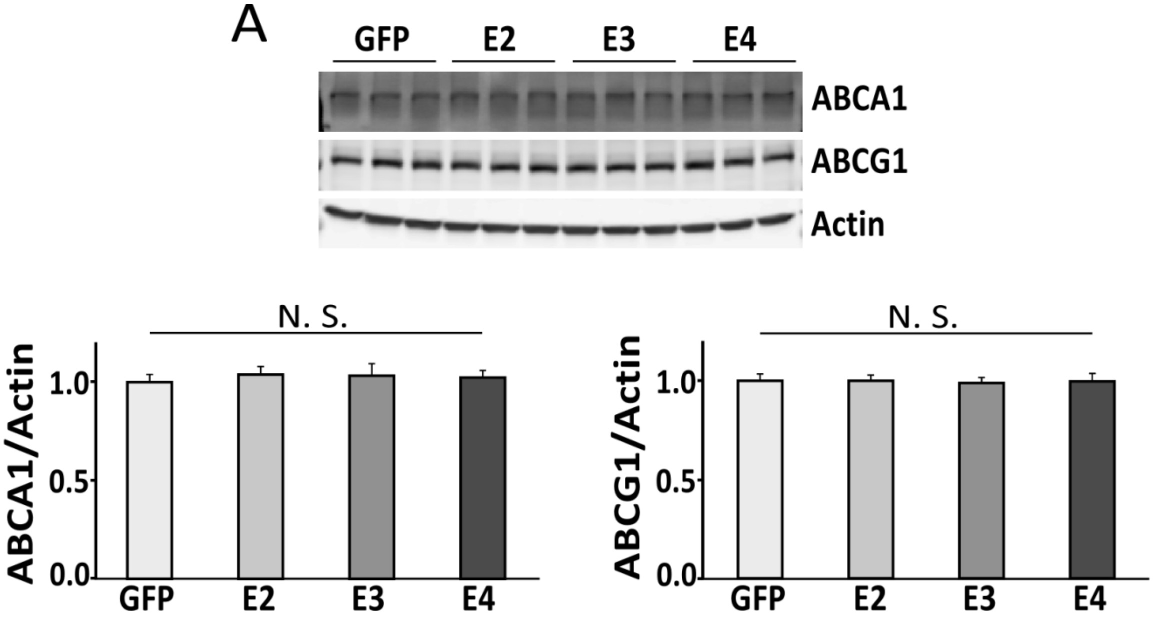
GFP
E2
E3
E4
ABCA1
ABCG1
Actin
N. S.
ABCA1/Actin
1.0
0.5
0.0
GFP
E2
E3
E4
N. S.
ABCG1/Actin
1.0
0.5
0.0
GFP
E2
E3
E4

B

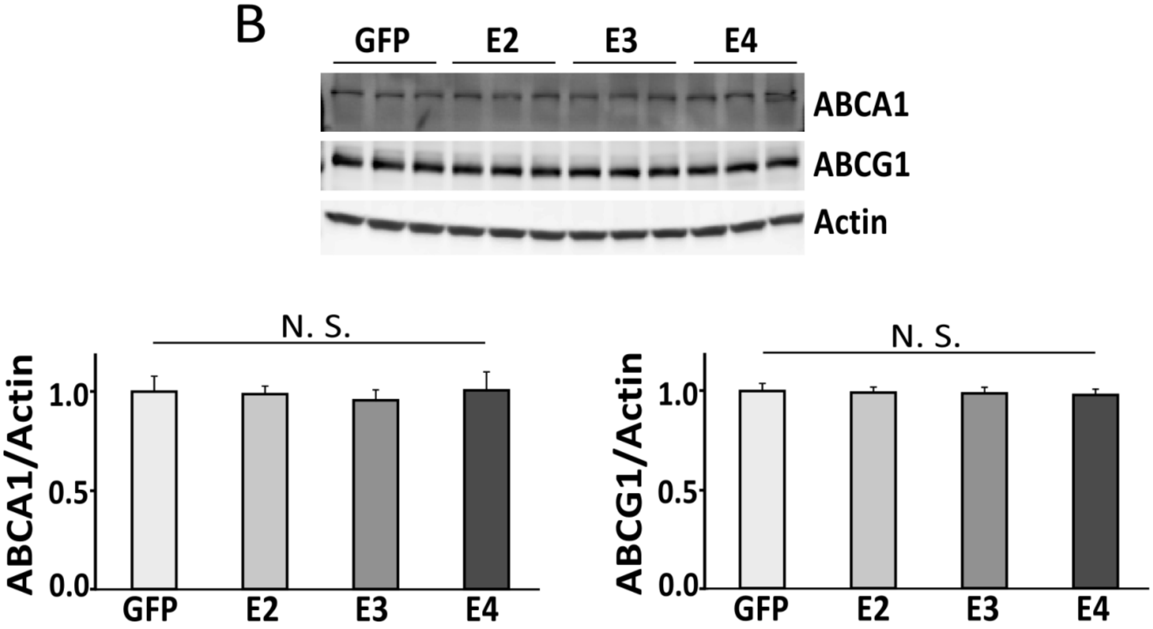
GFP
E2
E3
E4
ABCA1
ABCG1
Actin
N. S.
ABCA1/Actin
1.0
0.5
0.0
GFP
E2
E3
E4
N. S.
ABCG1/Actin
1.0
0.5
0.0
GFP
E2
E3
E4

Figure S3. Overexpression of apoE isoforms does not affect the levels of ABCA1 and ABCG1 in apoE-TR mice

Neonatal P2 apoE3-TR or apoE4-TR mice were injected with AAV viruses and brains were harvested as in Figure 2. Mouse brain cortical tissues were lysed in TBSX and 30 μg of protein lysates were loaded for Western blot. The levels of ABCA1, ABCG1 and actin from the brains of apoE3-TR mice (A) and apoE4-TR mice (B) were detected and quantified. Data are expressed as mean ± SEM (n=3-5). N.S., not significant.

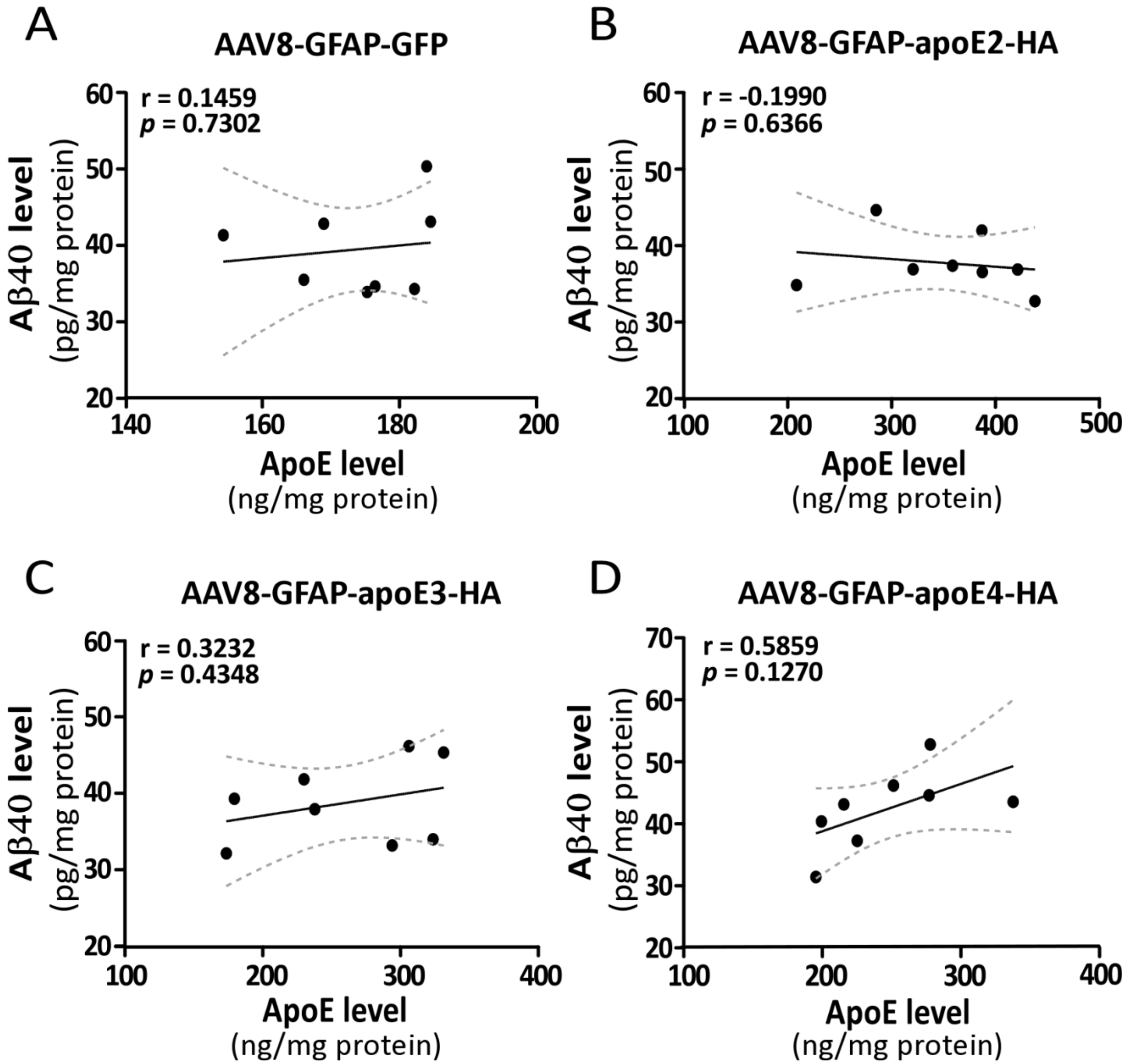
A
AAV8-GFAP-GFP
r = 0.1459
p = 0.7302
Aβ40 level
(pg/mg protein)
ApoE level
(ng/mg protein)
B
AAV8-GFAP-apoE2-HA
r = -0.1990
p = 0.6366
Aβ40 level
(pg/mg protein)
ApoE level
(ng/mg protein)
C
AAV8-GFAP-apoE3-HA
r = 0.3232
p = 0.4348
Aβ40 level
(pg/mg protein)
ApoE level
(ng/mg protein)
D
AAV8-GFAP-apoE4-HA
r = 0.5859
p = 0.1270
Aβ40 level
(pg/mg protein)
ApoE level
(ng/mg protein)

Figure S4. Correlation analysis between mouse endogenous Aβ and apoE in apoE3-TR mice transduced with viruses carrying AAV8-GFAP-apoE isoforms

Three months after intracranial delivery of viruses carrying AAV8-GFAP-GFP or AAV8-GFAP-apoE isoforms, the levels of mouse endogenous Aβ40 and apoE in the cortices of apoE3-TR mice were examined by Aβ40-specific ELISA and apoE-specific ELISA, respectively. (A-D) The correlation between levels of Aβ40 and apoE in the cortex of apoE4-TR mice transduced with GFP (A), apoE2 (B), apoE3 (C) or apoE4 (D) were analyzed and plotted. The Pearson correlation coefficient (r) and p values are shown.

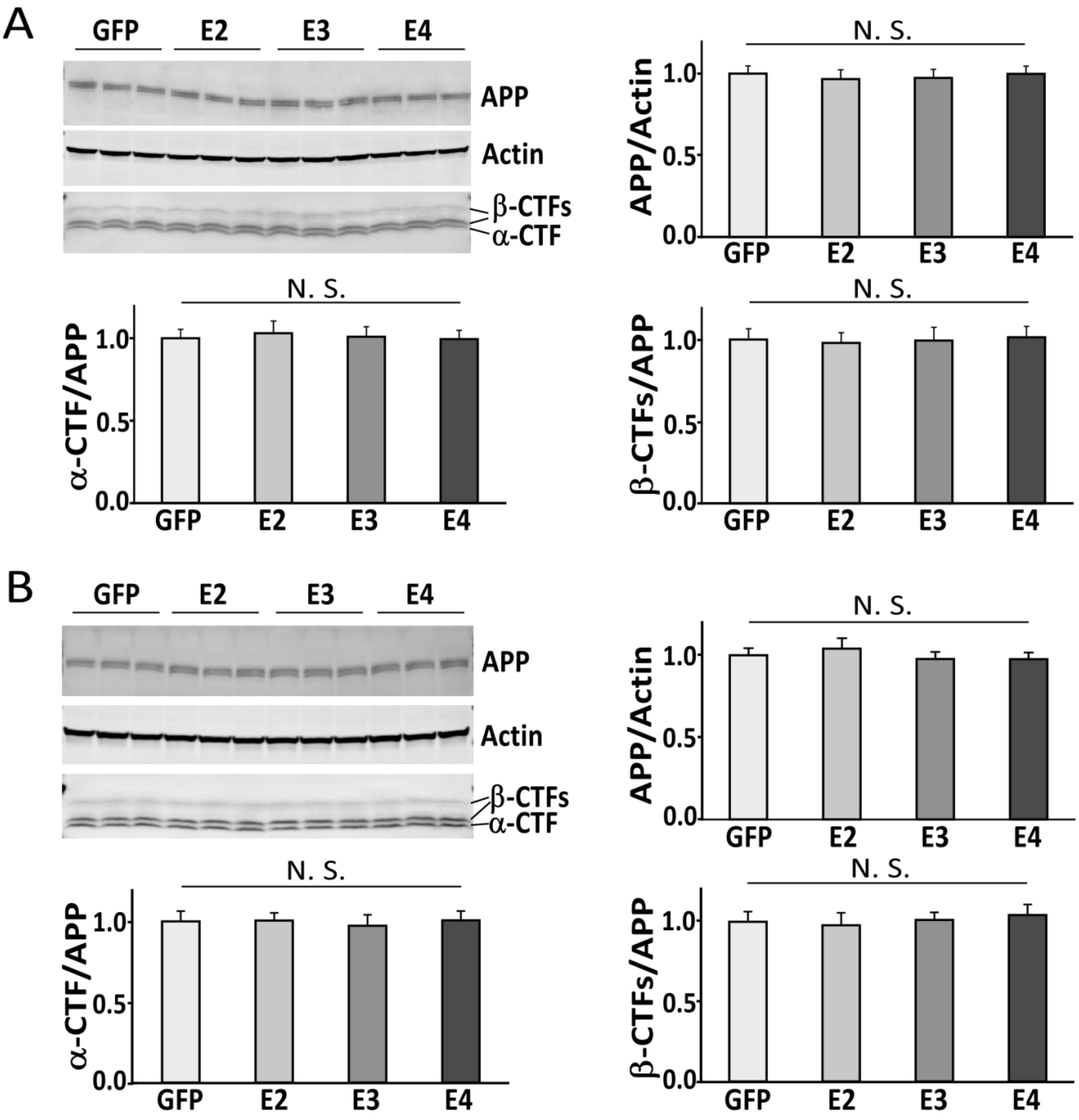
A
GFP
E2
E3
E4
APP
Actin
β-CTFs
α-CTF
N. S.
APP/Actin
α-CTF/APP
β-CTFs/APP
1.0
0.5
0.0
B
GFP
E2
E3
E4
APP
Actin
β-CTFs
α-CTF
N. S.
APP/Actin
α-CTF/APP
β-CTFs/APP

Figure S5. APP expression and processing are not altered in apoE-TR mice upon overexpression of apoE isoforms

Neonatal P2 apoE3-TR or apoE4-TR mice were injected with AAV viruses and brains were harvested as in Figure 2. Mouse brain cortical tissues were lysed in TBS containing 1% Triton X-100 (TBSX) and 30 μg of protein lysates were loaded for Western blott. The levels of APP and its C-terminal fragments, as well as β-actin from brains of apoE3-TR mice (A) and apoE4 TR mice (B) were detected and quantified. Data are expressed as mean ± SEM (n=3-5). N.S., not significant.
